# The Relationship between First Trimester 25-Hydroxyvitamin D3 Levels and Second Trimester Femur Length and Their Effects on Birth Weight and Length at Birth: A Preliminary Study

**DOI:** 10.1155/2019/3846485

**Published:** 2019-09-18

**Authors:** Elif Ganime Aydeniz, Umut Sari, Isil Tekin, Talat Umut Kutlu Dilek

**Affiliations:** ^1^Acibadem Mehmet Ali Aydınlar University Atakent Hospital Assisted Reproductive Techniques Unit, Istanbul, Turkey; ^2^Acibadem Mehmet Ali Aydınlar University Atakent Hospital Obstetrics and Gynecology Clinic, Istanbul, Turkey; ^3^Acibadem Mehmet Ali Aydınlar University School of Medicine, Istanbul, Turkey; ^4^Acibadem Mehmet Ali Aydınlar University School of Medicine, Department of Obstetrics and Gynecology, Istanbul, Turkey

## Abstract

**Objective:**

The main goal of our study was to assess relationships between first trimester 25-hydroxyvitamin D3 levels and infant birthweight and length at birth.

**Materials and Methods:**

We conducted a study over our medical records of 154 live-term births at Acibadem Atakent Hospital, Istanbul, Turkey. Subjects were classified into five independent groups.

**Results:**

We retrospectively reviewed a total of 154 live birth records. They took vitamin D3 supplement 1000 U/day. We classified the serum vitamin D levels into 5 groups by concentration. Group 1 comprised serum vitamin D levels <10 ng/ml (*n* = 41); group 2 comprised serum Vitamin D levels between >10–16 ng/ml (*n* = 33); group 3 comprised serum vitamin D levels >16–20 ng/ml (*n* = 26); group 4 vitamin D level between >20–30 ng/ml (*n* = 33) and group 5 comprised vitamin D levels >30 ng/ml. The femurs of infants were found to be longer between the groups, although the differences were not significant (*p*=0.054). There was also a statistically significant difference in the neonatal birth weight (*p*=0.048).

**Conclusion:**

We observed associations between low and high maternal 25-hydroxyvitamin D3 levels and fetal growth at birth weight but no difference in birth length. We conclude that we always need to conduct further research to be able to predict the effects of vitamin D deficiency.

## 1. Introduction

Vitamin D deficiency is a global health problem. Low vitamin D levels can be coexistent with preeclampsia, intrauterine growth restriction, small-for-gestational age sizes, skeletal problems, diabetes, and asthma. The effects of vitamin D levels on pregnancy bone mineralization and fetal growth are known. Controversies remain about the relationship between pregnancy serum 25-hydroxyvitamin D3 levels and neonatal weight. Two observational studies have reported a positive association [1]. The multiple ethnicities in the groups of both studies are a major limitation of their findings. The risk of neonatal vitamin D deficiency and the risk of lower birth weight are both increased by a maternal vitamin D deficiency [2]. Controversially, several studies have reported positive effects on birth length and birth weight. There is growing evidence that links vitamin D deficiency to immune system dysfunction, abnormal angiogenesis, and preeclampsia [3].

## 2. Objectives

In the first trimester, the fetus' daily accumulation of vitamin D in the skeleton is 2-3 mg, and in the last trimester, this rate doubles. Pregnant women's calcium absorption increases from early pregnancy but peaks in the third trimester. Research has shown that impaired placental development causes both abnormal angiogenesis and a decrease in the production of placental vitamin D and that low blood calcium levels are associated with hypertensive disorders [4]. A lot of studies have also shown that there is a communication between vitamin D levels and newborn size. A pregnant woman needs a daily intake of vitamin D of 800–1000 IU, but the actual need varies according to ethnicity, nutritional factors, and sunlight exposure [5]. It has also been shown that intrauterine bone hypomineralization is associated with vitamin D deficiency and then a reason for congenital rickets, craniotabes, and osteopenia [6]. Because of the possible effects on fetal somatic growth, our study's goal was to assess relationships between first trimester 25-hydroxyvitamin D3 levels and birthweight and infant birth length [7].

## 3. Materials and Methods

Our clinical retrospective review consists of the medical records of 154 live-term births from the Acibadem University of Mehmet Ali Aydınlar Atakent Hospital Department of Obstetrics and Gynecology Center, Istanbul, Turkey, between 2016 and 2018. The Ethical Committee of the Acibadem Mehmet Ali Aydinlar University Ethics Committee granted ethical approval. All the procedures we performed that involved human participants were carried out in accordance with the ethical standards of our institution and in accordance with the 1964 Helsinki Declaration and its later Amendments or with comparable ethical standards. Multiple pregnancies and pregnancies with previously known metabolic disorders were excluded from the study. Blood samples were collected in 12th to 14th weeks to measure 25-hydroxyvitamin D3. We used electro-chemiluminescence immunoassay (ECLIA) on the Roche Modular Analytics E170 (Roche Diagnostics, Mannheim, Germany) for serum 25-hydroxyvitamin D3 evaluation. The intra-assay coefficients of variation were 2.4% at 40.48 ng/mL.

Subjects were classified into five independent groups for first trimester serum vitamin D levels. Group 1 was comprised of serum vitamin D levels <10 ng/ml (*n* = 41); group 2 was comprised of serum Vitamin D levels between >10–16 ng/ml (*n* = 33); group 3 was comprised of serum vitamin D levels >16–20 ng/ml (*n* = 26); group 4 vitamin D level between >20–30 ng/ml (*n*: 33) and group 5 was comprised of vitamin D levels >30 ng/ml. We carried out our statistical analysis using MedCalc statistical analysis software version 12.3. The Kolmogorov–Smirnov test was used to assess the distribution of variables. The analysis of normally distributed continuous variables was done by the ANOVA test, and the analysis of non-normally distributed variables was done through the Kruskal–Wallis test. We considered the results statistically significant if the *p* value was less than 0.05.

## 4. Results

We conducted a retrospective review of the medical records of 154 live births.  Table 1 shows first trimester 25-hydroxyvitamin D3 levels (ng/ml), midtrimester femur lengths (mm), birthweights (g), and neonatal lengths (cm).

**Figure 1:**
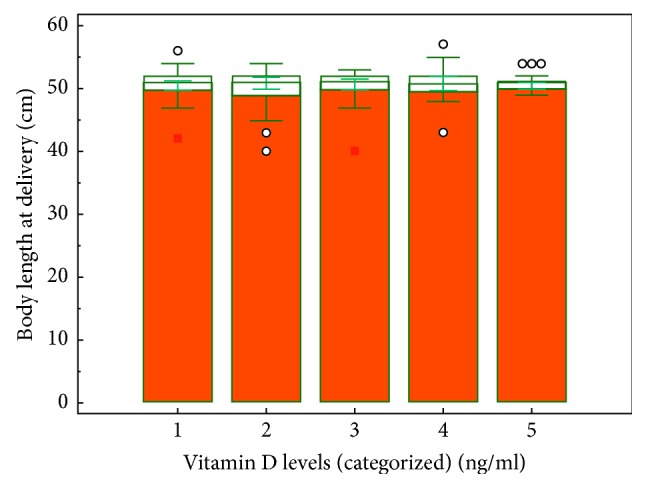
Relationship between vitamin D and body length of delivery.

47% of the deliveries were vaginal and 53% were by cesarean section (C-section). The median birthweight was 3287.5 g, and the median femur-length at birth 51 cm. The serum vitamin D levels were classified into 5 groups by concentration. Group 1 was comprised of serum vitamin D levels <10 ng/ml (*n* = 41); group 2 was comprised of serum vitamin D levels between >10–16 ng/ml (*n* = 33); group 3 was comprised of serum vitamin D levels >16–20 ng/ml (*n* = 26); group 4 vitamin D level between >20–30 ng/ml (*n*: 33) and group 5 was comprised of vitamin D levels >30 ng/ml. We measured second trimester femur lengths by ultrasound; and infant birthweight and birth length were compared for each of the five groups. The femurs of infants were found to be longer between groups, although the differences were not significant (*p*=0.054). (Figure 1).

**Figure 2:**
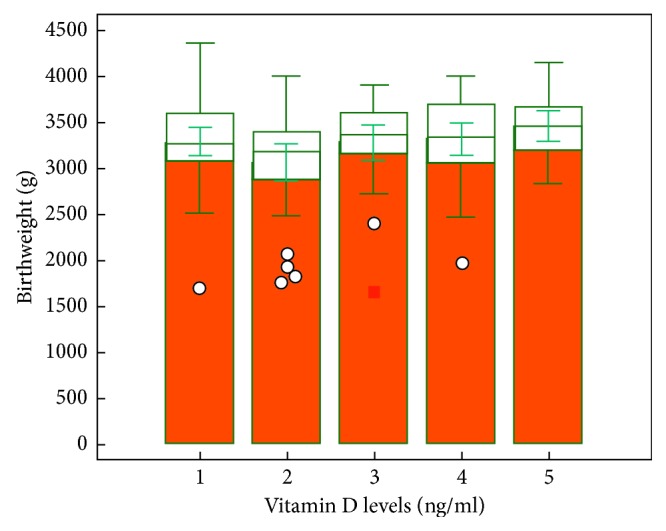
Relationship between vitamin D and birth weight.

Birthweights between the groups were statistically significant (Figure 2) There was also statistically significant difference in the neonatal birth weight (*p*=0.048).

## 5. Discussion

Vitamin D deficiency is a common global problem during pregnancy. The primary source of vitamin D is the exposure to sunlight. Studies have shown that serum 25-hydroxyvitamin D3 levels are very important chronic diseases such as diabetes, autoimmune disorders, and infections [8–10]. Sedentary indoor lifestyles, obesity, and avoiding sunlight each contributes to increased vitamin D deficiency. Pre-eclampsia is especially more prevalent when serum vitamin D levels are less than 10 ng/ml [10, 11]. Last studies showed that, in pregnant women, 25-hydroxyvitamin D3 serum level is not in a certain relationship with fetal growth in Europe and the United States [11]. The current evidence suggests there is no proof that maternal vitamin D levels affect bone formation in utero.

Growth occurs in femur at 34th week, and we can see it growing with ossification. Mahon et al. [11] developed a femoral splaying index (distal femoral CSA/FL ratio) by femur length (FL) and distal metaphyseal cross-sectional area (CSA).

All the pregnant women in our study took the multivitamin 1000 IU (25 *μ*g) D3 daily. Also, the pregnant women were not classified by open or closed clothes such as burka, seasonal variations, life habits (indoor life and regular walking), and feeding.

There is no study supporting the hypothesis that there is a link between vitamin D deficiency and impaired fetal growth. Another study reported that pregnant adolescents with serum 25-hydroxyvitamin D3 >50 nmol/L at delivery had higher fetal femur length (FL) and humeral length (HL) Z-scores at the 34th weeks. Also, a maternal calcium intake of less than 1050 mg/day was associated with lower FL and HL Z-scores. Both FL and HL Z-scores were higher in women with a sufficient calcium intake >1100 mg/d and 25-hydroxyvitamin D3 >50 mmol/L compared with the group with a combined calcium/vitamin D insufficiency (calcium intake <1100 mg/d and 25-hydroxyvitamin D3 <50 nmol/L) [12]. Fernandez-Alonso did not find any association between 25-hydroxyvitamin D3 levels and crown rump length (CRL) when using a routine first trimester ultrasound exam [13].

In our study, we compared vitamin D and fetal growth values in pregnant Turkish women, and we evaluated each of the growth parameters by ultrasound screening between weeks 22 and 24^t^ of pregnancy. We determined the first trimester maternal serum 25-hydroxyvitamin D3 and femur length generally in the 22nd week of pregnancy (though sometimes in the 21st or 23rd weeks) and compared these values by birth weight and birth femur length. There was no relationship between vitamin D levels and neonatal birth weight and length in the first trimester. Although we did not follow up any relations between pregnant serum vitamin D and baby development, infant birth weight, and length at birth in our study, the outcomes of our study should not be interpreted as that serum vitamin D levels are not an important determinant during pregnancy, until future studies provide new data. Our study had some limitations, such as its cross-sectional design and limited sample size. Future studies should encompass more parameters, such as the woman prebirth body mass index, height, weight, and weight gain during pregnancy.

## 6. Conclusion

Next studies are necessary to define the role of vitamin D and calcium supplementation for fetal growth and neonatal birth weight. We did not observe statistically significant differences between maternal 25-hydroxyvitamin D3 levels and fetal birth length. We conclude that we need to conduct further research to be able to predict the effects of vitamin D deficiency on pregnancy and the newborn.

## Figures and Tables

**Table 1:** Comparison of 5 different groups by vitamin D3 levels.

	Group 1	Group 2	Group 3	Group 4	Group 5	*p* value^*∗∗*^
Femur length (mm)^*∗*^	35.19	34.86	34.87	33.4	35.87	0.054
Birth weight (g)^*∗*^	3292.8	3073.93	3279.57	3326.51	3469.52	0.048
Birth length (cm)^*∗*^	50.52	50.29	50.5	50.97	51	0.79

^*∗*^Expressed as mean. ^*∗∗*^*p* < 0.05, statistically significant.

## Data Availability

Our data table has been deposited in figshare at DOI: 10.6084/m9.figshare.7679621.
